# Aggregation Methods for Quantifying PTM and Structural Changes in Bottom-Up Proteomics

**DOI:** 10.1021/acs.jproteome.5c00782

**Published:** 2026-05-15

**Authors:** Erik D. VonKaenel, Jordan C. Rozum, Tong Zhang, Kelly G. Stratton, Lisa M. Bramer, H. Steven Wiley, Wei-Jun Qian, Amy C. Sims, John T. Melchior, Song Feng

**Affiliations:** † Biological Sciences Division, 6865 Pacific Northwest National Laboratory, Richland, Washington 99352, United States; ‡ Environmental Molecular Sciences Division, Pacific Northwest National Laboratory, Richland, Washington 99352, United States; § Nuclear, Chemical, and Biological Technologies Division, Pacific Northwest National Laboratory, Richland, Washington 99352, United States

**Keywords:** bottom-up proteomics, limited-proteolysis mass spectrometry, post-translational modification, proteomics quantification

## Abstract

Bottom-up proteomic workflows rely on sequential preprocessing steps, commonly including peptide-to-protein aggregation (“roll-up”), to enhance data reliability and interpretability. While roll-up is effective for protein-centered analyses, it may be suboptimal for applications focused on post-translational modifications (PTMs) or protein structural changes, such as limited proteolysis–mass spectrometry (LiP-MS). Here, we investigate how different roll-up strategies influence site-level quantification in PTM differential analysis. Moreover, we introduce a novel site-centric roll-up approach tailored for LiP-MS, which quantifies proteolytic fragments rather than solely tryptic peptides. We benchmark these methods through simulation studies, comparing their sensitivity and specificity in detecting structural and PTM-driven changes. We found that the *median* and *mean* roll-up methods outperform the *sum* method in both PTM and LiP proteomics, and site-level quantification in LiP outperforms peptide-level quantification. Our findings offer the first systematic, data-driven guidance for selecting roll-up techniques in site-level proteomic analyses, with implications for both PTM-focused and structural proteomics studies.

## Graphical abstract

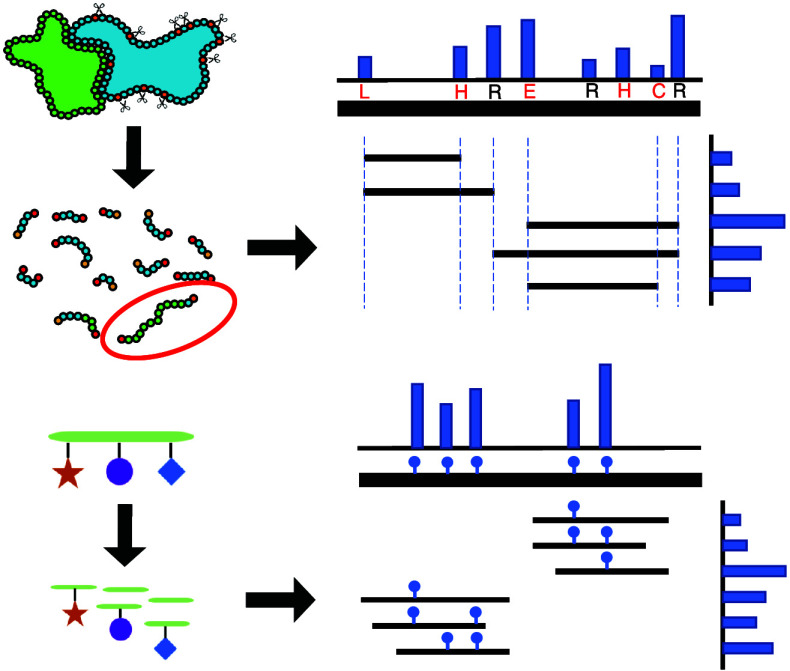

## Introduction

Modern advancements in experimental workflows and computational pipelines now support biomarker discovery, mechanistic studies of disease pathways, and therapeutic target validation through simultaneous quantification of thousands of proteins. During these advancements, bottom-up proteomics remains the predominant strategy for large-scale protein analysis, leveraging enzymatic digestion (typically with trypsin) to convert denatured proteins into measurable peptides.^1,2^ While this approach circumvents mass spectrometry’s limitations in analyzing intact proteins – including structural heterogeneity and dynamic range constraints – it introduces computational challenges in deriving biological insight from peptide signatures.^3^ Though it is possible to analyze peptide signatures directly,^4–6^ it is often desirable to reconstruct protein-level information from peptide data. Critical to this process are “roll-up” algorithms that aggregate peptide intensities while accounting for shared sequences, ionization efficiencies, and missing values.^7,8^ Optimization of these aggregation strategies remains underexplored in PTM analysis and structural proteomics.

Method selection in quantitative proteomics is fundamentally dependent on the biological question being addressed. Studies seeking changes in the abundance of bulk proteins typically employ label-free quantification or isobaric tagging, while PTM-focused investigations require enrichment techniques and site-specific quantification. Similarly, emerging methods such as limited proteolysis mass spectrometry (LiP-MS)^9–12^ demand tailored workflows: this technique employs partial digestion with proteases (e.g., proteinase K) under native conditions to probe structural conformations, followed by complete tryptic digestion after denaturation. Such method-specific requirements highlight the need for optimized data processing strategies that align with each technology’s analytical goals.

Our study focuses on two critical applications where peptide aggregation strategies warrant reevaluation: (1) PTM analysis that requires site-level quantification and (2) LiP-MS workflows that detect structural changes. Current roll-up methods are designed for protein-level quantification, where many peptides are mapped to a protein. There is no study assessing whether roll-up methods capture site-specific modification dynamics. Similarly, LiP-MS introduces unique analytical challenges: its stage-one digestion patterns reflect tertiary structure rather than sequence abundance, necessitating specialized approaches to distinguish conformational changes from expression-level variation.

We present a systematic evaluation of roll-up strategies using ground-truth simulation frameworks for both PTM and LiP-MS applications. For PTM analysis, we compared traditional protein-level aggregation with site-specific models that account for modification stoichiometry and peptide detectability. In LiP-MS analysis, we contrast protease cleavage site aggregation with direct tryptic peptide analysis to determine optimal strategies for structural change detection. Recent independent work advocates rolling-up the quantification of LiP-MS to “cut-site” level.^13^ However, there is yet no rigorous or comprehensive assessment of LiP-MS roll-up method fidelity in recovering changes to protein surface exposure using ground-truth data. We address this current need using simulations that use a well-defined ground truth and incorporate real-world variability factors including digestion efficiency bias, ionization competition effects, and modification site occupancy rates.

This work makes three key contributions: First, we establish computational guidelines for site-level roll-up in PTM studies, demonstrating improvements in true positive rates compared to protein-centric methods when modification stoichiometry varies independently of abundance. Second, we validate protease site aggregation as the optimal strategy for LiP-MS, achieving higher accuracy of structural change detection compared to using tryptic peptides alone. Third, we provide an open-source simulation framework (https://github.com/PNNL-Predictive-Phenomics/OptRollingup) that models both modification dynamics and structural perturbations, enabling method developers to test aggregation strategies under controlled conditions. These findings advance quantitative proteomics by aligning data processing methodologies with the specific biological features each experimental approach aims to capture.

## Methods

We systematically evaluated peptide aggregation methods for site-level quantification in PTM and LiP-MS proteomics by developing two simulation frameworks that incorporate known abundance patterns. This section describes (1) the mathematical foundation of roll-up operations, (2) the protocols used to generate data sets, and (3) the metrics employed to compare aggregation approaches under controlled variability conditions.

Following current convention, we use the term “rolling-up” or “roll-up” to denote the process of aggregating measured peptide abundances to a higher level object (Figure 1). We operationalize roll-up as a two-stage computational process applied to peptide raw intensities: the first stage is intensity scaling, where raw peptide abundances are normalized to account for technical variability; then the second stage is feature aggregation, where scaled intensities are combined using statistical estimators (*mean*, *median*, or other robust regression models) to infer higher-order biological entities - proteins for conventional analyses, modification sites for PTMs, or protease cleavage regions for LiP-MS.

**Figure 1:**
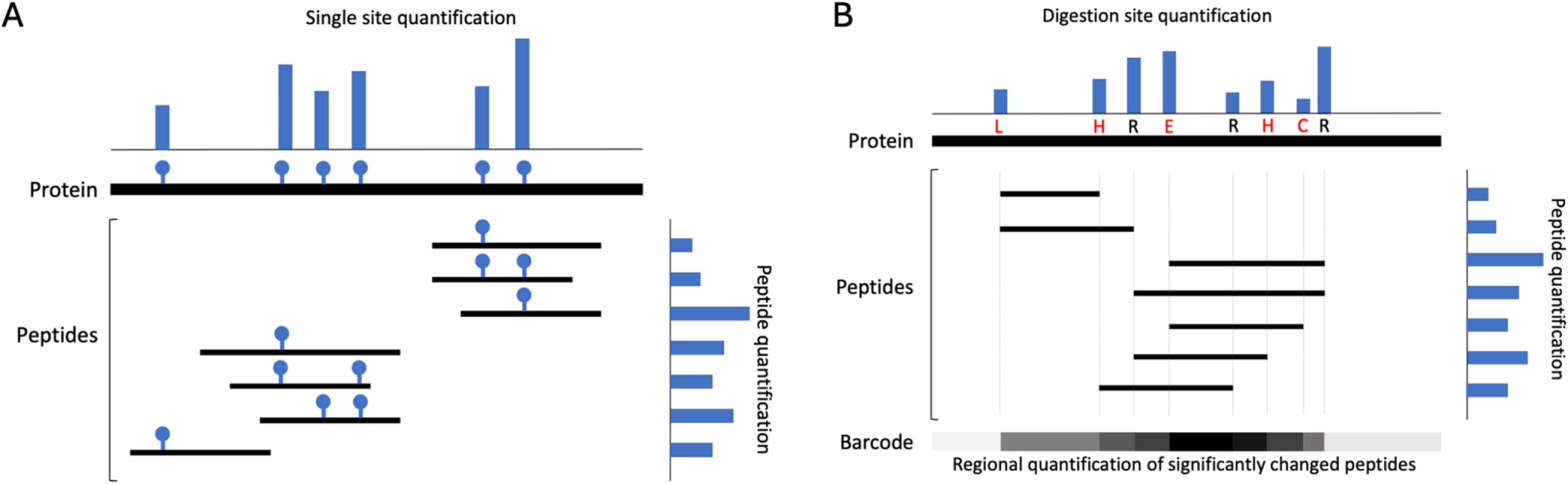
Illustration of roll-up quantification of PTM (A) and LiP (B) proteomics data from peptide to site level. In PTM proteomics, the goal is to quantify relative differences in PTM site abundance between experimental groups, while in LiP proteomics, the goal is to quantify structural changes in proteins across groups. The roll-up methods considered in this work are applied to the peptide abundances to arrive at a site-level quantification. These methods differ in how they scale peptide quantification and in what aggregation function (mean, median, sum) they apply to peptides corresponding to each site.

### Roll-Up Methods for PTM Proteomics

In PTM proteomics, the primary aim is to quantify relative changes in PTM site abundance across experimental groups. For example, one might test whether the log_2_ fold change of serine phosphorylation differs from zero when comparing a treatment to a control. Although this resembles a standard proteomics inference task, the focus here shifts from proteins to site modifications. It remains unclear, however, which roll-up strategy most accurately and robustly captures the true fold change. Several popular aggregation methods for protein-level analyses – implemented in the pmartR^14^ package – can be adapted for site-level applications. Specifically, we considered the three scaling methods (*rollup*, *rrollup*, and *zrollup*) in combination with three aggregation methods (mean, median, and sum). In these approaches, *rollup* aggregates all peptides mapping to the site without scaling, *rrollup* ^15^ scales each peptide by the abundance of the most frequently observed peptide, and *zrollup* ^16^ scales peptides based on their estimated standard error across all peptides mapping to the same site. The popular MaxQuant^7^ software implements an optional PTM site-level rollup^17^ (by default it selects a representative peptide without rolling up); the method implemented is equivalent to the *rollup* method here with the sum aggregation function.

### Simulating PTM Proteomics to Generate Synthetic Data

We adopt a direct strategy to simulate post translational modifications by explicitly choosing sites for each simulated protein to label as “modified”, and counting the abundance of each modification site for each group. We apply a statistical model for selecting modification sites and use previously published methods for simulating the digestion of amino acid chains to generate a distribution of peptides. Finally, we apply an observation model to account for measurement effects. In the remainder of this subsection, we describe these steps in greater detail.

We consider a protein as an amino acid sequence of length *Q*, which has Q* ≤ *Q* potential modification sites, that is, amino acid locations in the sequence. Then a modified protein contains *R* ≤ Q* *modification identifiers* at distinct sites in the sequence. Since a sequence can be represented by letters, each denoting a specific amino acid, it is standard for modification identifiers to be denoted by a special character, say, “#”. Without loss of generality, we establish the potential modification sites by limiting the possible PTM sites to the amino acid serine.

Let *M* be the absolute abundance of the sequence. For each site *r* ∈ {1, ..., *R*}, we uniformly sample *m*_*r*_ ≤ *M* of the *M* total sequences without replacement to modify at site *r*. Specifically,

1 $$m_{r}∼U\left(1,M\right)$$

where *U*(*a*,*b*) is the uniform distribution on {*a*, ..., *b*}. We let *m*_*r*_ represent the true abundance of a modification at site *r*. This can be operationalized as an *M* × *R* binary matrix *A*, where *A*_*m*,*r*_ = 1 indicates protein *m* was modified at site *r*, and *A*_*m*,*r*_ = 0 indicates protein *m* was not modified at site *r*. Each of the *M* replicates for a protein sequence are then modified by injecting a “#” directly into the sequence at each modification site, and then the modified proteins are artificially digested into (potentially) modified peptides.

Artificial digestion using trypsin is performed using the OrgMassSpecR^18,19^ package. This software simulates perfect trypsin digestion, so we make a second pass during artificial digestion to randomly merge adjacent peptides, thus simulating an imperfect digestion. Missed cleavage sites are randomly sampled with probabilities proportional to the adjacent peptide lengths: shorter peptides are merged with higher probability than larger ones. We control the proportion of missed cleavage sites using a parameter in the simulation. For digestion by trypsin, we impose the rule that the cleavage site must be a Lysine or Arginine that is not preceded by a Proline. These peptides are counted directly to arrive at the true abundance for each unique, potentially modified peptide.

We model both site-specific and subject-specific effects in the observed abundance of each modified peptide. The *site effect* captures how a modification at position *r* alters the peptide’s *m*/*z*-derived abundance. Concretely, let *p* denote the abundance of an unmodified peptide, and let *S* ⊂ {1, ..., *R*} be the set of modification sites on that peptide; we write *p*_*S*_ for the abundance of the peptide carrying exactly those modifications. Next, suppose there are *K* experimental groups (*k* = 1, ..., *K*), and each group *k* contains *N*_*k*_ subjects. For each site *r* in group *k*, let *m*_*r*,*k*_ denote the site-specific shift in abundance. We then add both the appropriate *m*_*r*,*k*_ terms (one for each *r* ∈ *S*) and a subject-specific offset to obtain the final abundance of every modified peptide, as follows:

2 $${\mathrm{p̃}}_{i,k,S}={p_{S}}^{*}2^{\beta_{\mathrm{ik}}*}\prod_{r\in S}2^{\alpha_{r}}$$

where *i* denotes the subject. This results in a linear modification of the log_2_ abundances

3 $$\log_{2}{\mathrm{p̃}}_{i,k,S}=\log_{2}p_{S}+\beta_{\mathrm{ik}}+\sum_{r\in S}\alpha_{r}$$

The subject effect acts as a random subject effect in a standard random effects model and is sampled via

4 $$\beta_{\mathrm{ik}}∼N\left(0,\sigma_{\mathrm{subj}}\right)$$

and similarly, the site effect is sampled via

5 $$\alpha_{r}∼N\left(0,\sigma_{\mathrm{site}}\right)$$

After generating a full simulated data set – comprising multiple proteins, subjects, and experimental groups – we introduce artificial missingness following the approach of Bramer et al.^20^ In particular, we emulate the missing-data pattern characteristic of isobaric-labeled proteomics (the *labeled* case). First, each subject is randomly assigned to a synthetic TMT plex. Then, to create missing values, we pick peptides for omission with probability inversely proportional to their abundance; once selected, that peptide’s measurements are removed for every subject in one randomly chosen plex. Because our simulation already includes some missingness due to imperfect digestion, we set a predetermined overall missingness rate for the data set and cease deleting observations once that target level is reached.

Because we know the true, simulated abundance of each modification site, we can calculate the exact fold change between groups under each condition. This ground truth lets us directly assess how well different roll-up methods reconstruct group-level differences when aggregating site-level measurements in bottom-up proteomics.

### Evaluation of the Aggregation in Quantifying the PTM Changes

To evaluate each PTM site-level roll-up method, we leverage the known true abundance log_2_ fold-change (log_2_ FC) for every PTM across groups. For each simulated data set, we calculate the root mean squared error (RMSE) of log_2_ FC at each site for every protein. Within each simulation scenario, we then average the site-level RMSEs across all data sets to obtain a mean RMSE for each method, and we report that mean alongside its standard error across replicates.

We explore several simulation scenarios, with a primary focus on how missing data affects site-wise quantification. Specifically, we impose three levels of global missingness: 0, 25, and 50%. To keep computations manageable, each synthetic data set contains either 5 or 10 randomly chosen protein sequences; although this number is small relative to real proteomes, increasing the count did not appreciably change the comparative performance of the methods. We fix the total abundance *M* for each protein at either 100 or 250 – values lower than typical real-world abundances but shown to have minimal impact on our simulation outcomes. Similarly, we simulate proteins of length 100 or 200 amino acids, since varying sequence length beyond these did not materially affect results. For both site-level and subject-level effects, we test three variance levels: 0, 1, and 9. Finally, we introduce incomplete digestion at three rates – 0, 25, and 50% of possible cleavages – so as to mimic different degrees of proteolytic inefficiency.

### Roll-Up Methods for LiP-MS

LiP analyses focus on detecting conformational changes in proteins by comparing their structural distributions across groups. Traditionally, this involves examining tryptic peptides – those whose sequences begin and end at a trypsin cleavage site – since regions of the protein that were protected from the first (proteinase K) digestion stage will yield higher relative abundances of tryptic peptides. For example, if 50% of replicates in group 1 adopt conformation A but only 10% of replicates in group 2 do, one would expect a notable fold change in the corresponding tryptic peptides between the two groups. However, because tryptic peptide abundances can fluctuate due to variability in digestion or structure, relying solely on them can be unstable. Moreover, this standard approach effectively discards any peptides that are not fully tryptic.

Instead, we propose a site-level roll-up strategy that aggregates peptide intensities around proteinase K cleavage sites (ProK site). Concretely, for each cleavage site, we collect all peptides immediately upstream and downstream of that site, then summarize them (using sum, mean, or median). By pooling multiple peptides per site and performing inference on these site-level aggregates, we aim to reduce the random variation that plagues tryptic-peptide analyses.

### Simulating LiP-MS to Generate Synthetic Data

To mimic LiP *in silico*, we simulate a two-stage digestion process. First, we mask certain regions of the amino acid sequence to represent folded (protected) segments. Only those unmasked regions are accessible to a simulated proteinase K digest. After this first digestion, we unmask the entire sequence (simulating denaturation) and then perform a simulated trypsin digest following the same procedure outlined in the section Simulating PTM Proteomics to Generate Synthetic Data.

The masking step proceeds as follows: for each protein *P* (with sequence *a*_1_ *a*_2_ *a*_3_...), we select *q* nonoverlapping masked regions *M*_*i*_ by traversing the sequence from start to finish. We alternate between masked segments *M*_*i*_ and unmasked gaps *G*_*j*_, so that

$$P=G_{1}M_{1}G_{2}M_{2}...\mathrm{with}\left|G_{j}\right|∼\mathrm{Pois}\left(\lambda_{G}\right),\left|M_{i}\right|∼\mathrm{Pois}\left(\lambda_{M}\right)$$

where |·| denotes the number of amino acids. In practice, we generate each *G*_*j*_ and *M*_*i*_ by drawing their lengths from independent Poisson distributions with means λ_*G*_ and λ_*M*_. Within a given experimental group, we assign each masked region a specified proportion between 0 and 1. Then, for each replicate, we randomly decide whether that region remains masked or becomes unmasked until the target proportion is reached. Thus, each group has a known distribution of folded (masked) versus unfolded (unmasked) regions.

For each folded protein (i.e., with masked regions), we perform a simulated proteinase K digest that cleaves immediately after any aliphatic, aromatic, or hydrophobic residue – except that cleavage cannot occur within a masked region. That is, we allow cleavage at Alanine, Valine, Leucine, Isoleucine, Phenylalanine, Tyrosine, Tryptophan, Methionine, and Proline residues. We first simulate a perfect proteinase K digest, then merge adjacent small peptides into larger ones to mimic an imperfect digestion, analogous to our PTM simulation strategy. The result is a set of proteinase K peptides that faithfully reflect limited proteolysis with masked regions intact.

After this first-stage digest, we simulate denaturing the protein by removing all masks. We then perform a simulated trypsin digest: first with an ideal (perfect) cleavage, followed by merging of small peptides to replicate real-world inefficiencies. This two-stage procedure yields tryptic peptides that depend on whether certain regions were masked during the proteinase K digest step.

To generate data across multiple groups, we proceed as follows: (1) Draw an artificial amino acid sequence for each protein, sampling residues either from an empirical distribution (if available) or uniformly over the 20 standard amino acids. (2) For each group, sample a masking pattern as described above, assigning each masked region a group-specific prevalence (a proportion between 0 and 1). Then, for each replicate within that group, randomly mask or unmask each region until the group-level masking proportions are satisfied. Consequently, we have a known differential distribution of masked (folded) regions across groups. (3) Introduce subject-level noise by allowing random deviations in the simulated digestion steps (both proteinase K and trypsin), so that each replicate’s peptide abundances vary around the values set as ground truth.

Because LiP experiments are usually label-free,^10^ we simulated label-free missingness. This follows the same logic as the labeled case – except that, instead of deleting all measurements within a plex, we randomly remove data from individual subjects. We continue omitting observations until a prespecified global missingness threshold is reached.

### Evaluation of the Aggregation in Quantifying the Structural Changes

Consider the comparison of a group of replicates (group 2) that share a treatment condition to a baseline group of replicates (group 1) that share a different treatment condition (e.g., a control group). In the site-wise roll-up approach, a masked region more prevalent in group 2 yields a positive expected log_2_ fold change; if the masked region is more prevalent in group 1, the expected log_2_ fold change is negative. The magnitude of this expected fold change is the log_2_ ratio of the known masking proportions injected into each group. If a region is masked equally (or not at all) in both groups, the expected log_2_ fold change is zero.

In the tryptic peptide approach, we compute the observed fold change of each tryptic peptide, then aggregate these within each masked or unmasked region using the median; for the site-wise method, we take the proteinase K peptide abundances rolled up to each cleavage site, then aggregate those site-level values within each masked or unmasked region (again using median). We then assess each method’s ability to detect a region that is masked in only one group (indicating structural differences) versus regions that are masked or unmasked equally (indicating no structural difference).

We vary the following parameters to explore their effects on performance of aggregation methods: sample size per group (5, 10, 25, or 50), protein copy number (100, 500, or 1000), missingness ratio of tryptic cleavage sites (0, 25, or 50%), missingness ratio of ProK sites (0, 25, or 50%). While we explored these parameters by varying them combinatorically, we fixed the protein length as 1000 amino acids and set the means for the gap and masked-region lengths λ_*G*_ = 25 and λ_*M*_ = 50, respectively. The minimum tryptic sites per masked region are set at least 5.

In an additional simulation, we fix the samples per group (25), replicates per protein (500), and missed-cleavage rates (25% each for trypsin and proteinase K), but vary λ_*G*_ and λ_*M*_ over {10, 50, 100} and {25, 50, 200}, respectively. This tests whether different masked-region sizes and distributions change which analysis method (tryptic versus site-wise) performs best. In this experiment, we use only median aggregation for site-level roll-up.

## Results

### Simulations Recapitulate Quantitative PTM and LiP Proteomics

To comprehensively assess roll-up strategies, we conducted two large-scale simulation studies – one focused on PTM quantification and the other on LiP-MS structural analysis – spanning a wide range of biologically relevant parameters. In the PTM simulations, we generated data sets varying: protein sequence length, protein abundance, global missingness rates, missed-cleavage rates, and number of proteins, etc.

In total, these choices yielded over 600 distinct parameter combinations, each replicated multiple times to ensure robust estimates of performance. For each simulated data set, we computed the true site-level log_2_ fold changes (log_2_ FC) and then applied nine combinations of scaling (*rollup*, *rrollup*, *zrollup*) and aggregation (*mean*, *median*, *sum*) to quantify their accuracy via the root-mean-squared error (RMSE) across all modification sites (see Methods).

Similarly, our LiP-MS simulations explored: sample sizes per group, protein copy number levels, missed-cleavage rates for both proteinase K and trypsin, masked-region length (λ_*M*_) and gap length (λ_*G*_). We compared two inference strategies: (1) traditional tryptic-peptide aggregation, summarizing log_2_ FC of fully tryptic peptides within masked vs unmasked regions; and (2) our proposed site-level roll-up at ProK sites, aggregating peptide intensities immediately upstream and downstream of each site. Performance was again evaluated by RMSE against the known log_2_ FC across masked regions.

### Aggregation of Site-Level Modifications Quantifies PTM Changes

#### *Mean* and *Median* Methods Outperform *Sum* Aggregation

Across all simulation scenarios we observed that *rollup* and *rrollup* scaling methods yield substantially lower errors compared to *zrollup* (median RMSE values ≈ 0.35–0.45 versus >0.70); see Figure 2. Among aggregation functions, mean and median perform nearly identically (median RMSEs within 0.02 of each other), while sum aggregation produces the highest errors (median RMSE ∼ 1.10), reflecting its sensitivity to outlier peptides and missingness.

**Figure 2:**
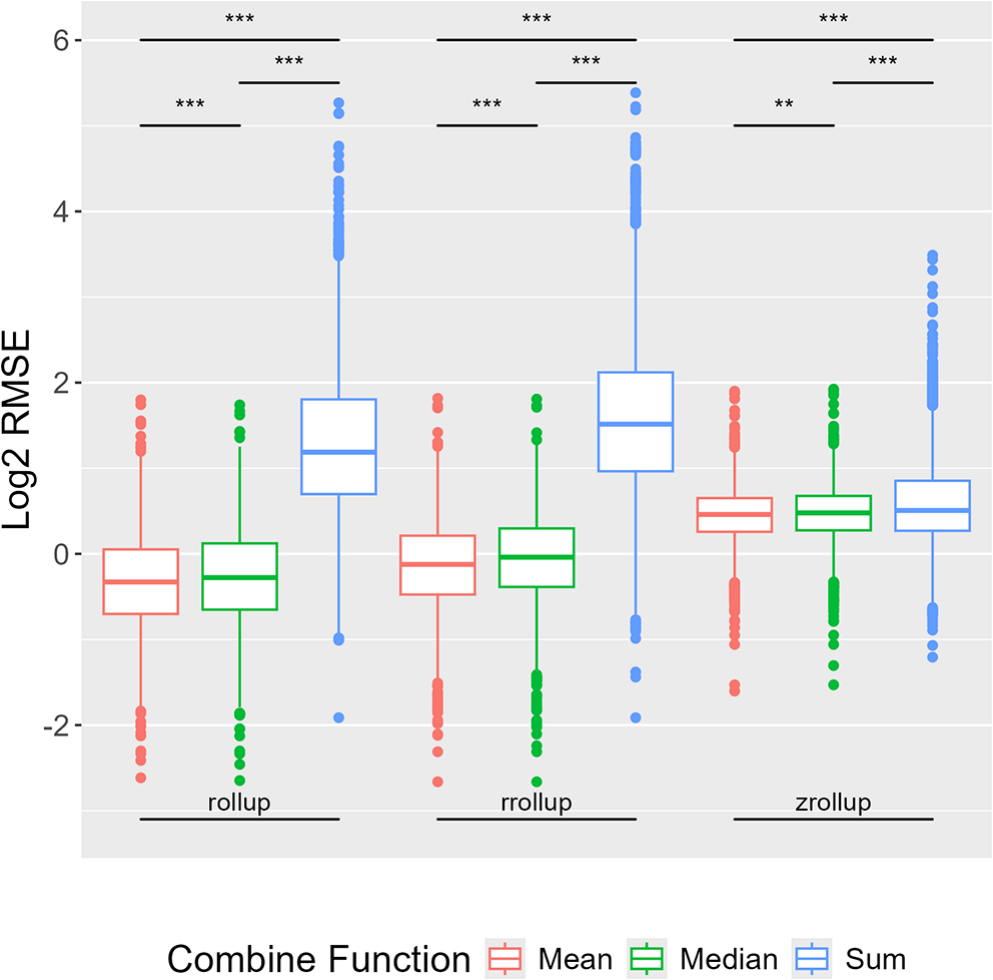
Aggregated PTM simulation results for each rollup method show different levels of accuracy depending on the function used to combine peptides. The box-plots show the RMSE distributions from multiple simulation instances for different roll-up methods. The colors represent roll-up methods with *mean*, *median*, and *sum*, while the *x*-axis is showing different scaling before rolling-up (see Methods). Due to the high number of simulation runs, all differences in the means are significant at the 0.01 level (**) or 0.001 level (***) by Student’s *t* test.

#### Site-Level Roll-Up is Robust to Missingness and Variability

We next quantified the effect of the missingness ratio of peptides, protein abundance and length, and missed cleavage ratios (see Methods). With broad combinatorial parameter sweeping, we found that the performances of all aggregation methods in quantifying the PTM changes are robust (Supporting Figures S1, S2, S3). Across 0, 25, and 50% missingness levels, *rollup*-mean and *rrollup*-median maintain low RMSEs (<0.50) even under extreme data loss, whereas *zrollup* methods degrade markedly (RMSE > 0.90 at 50% missingness). Increasing subject-level variance (σ_subj_) from 0 to 9 broadens the RMSE distributions by ∼0.10 but does not alter the rank ordering of methods. Similarly, peptide digestion inefficiency (missed cleavage up to 50%) and changes in protein abundance (M = 100 vs 250) have minimal impact on relative performance, indicating that mean/median aggregation paired with *rollup* or *rrollup* scaling is generally optimal across realistic proteomics conditions. Detailed scenario-by-scenario results are provided in the Supporting Information.

Overall, the *rollup* and *rrollup* scaling methods delivered nearly identical performance, both substantially outperforming the *zrollup* approach. In terms of aggregation, mean and median produced similar accuracy and surpassed the sum. Between *rollup* variants, *rollup* achieved marginally lower RMSE, whereas *rrollup* exhibited slightly less variability across scenarios, indicating greater consistency.

### Aggregation of Site-Level Exposure to Quantify Structural Changes

We simulate a single protein with four differentially masked regions (highlighted in yellow). For each region, the injected masking ratio between groups 1 and 2 is either 2:1, 1:2, or 1:1, yielding expected log_2_ FC of −1, +1, or 0, respectively. For all the peptides digested from the protein, we aggregate the ion intensities from tryptric peptides sharing same the same tryptic site to represent the quantification of tryptic peptides changes and aggregating ion intensities from peptides sharing the same proteinase K digested sites to represent exposure level at proteinase K digestion sites. Then we calculate the log_2_ FC between the treatment and control groups at individual digestions sites. This resulting a series of digestion site changes along the protein sequence (Figure 3). In the synthetic data, we can observe more dramatic changes in masking regions, where treatment causes exposure changes, compare to the unmasked region. It shows that the synthetic data faithfully simulated the LiP-MS data. Then, we compared the true log_2_ FC at the residue level in both the masked and unmasked regions (dashed lines), as well as the quantification aggregated at residue level with the ground truth log_2_ FC from the synthetic data. We average log_2_ FC to compare the log_2_ FCs of each region (Figure 4).

**Figure 3:**
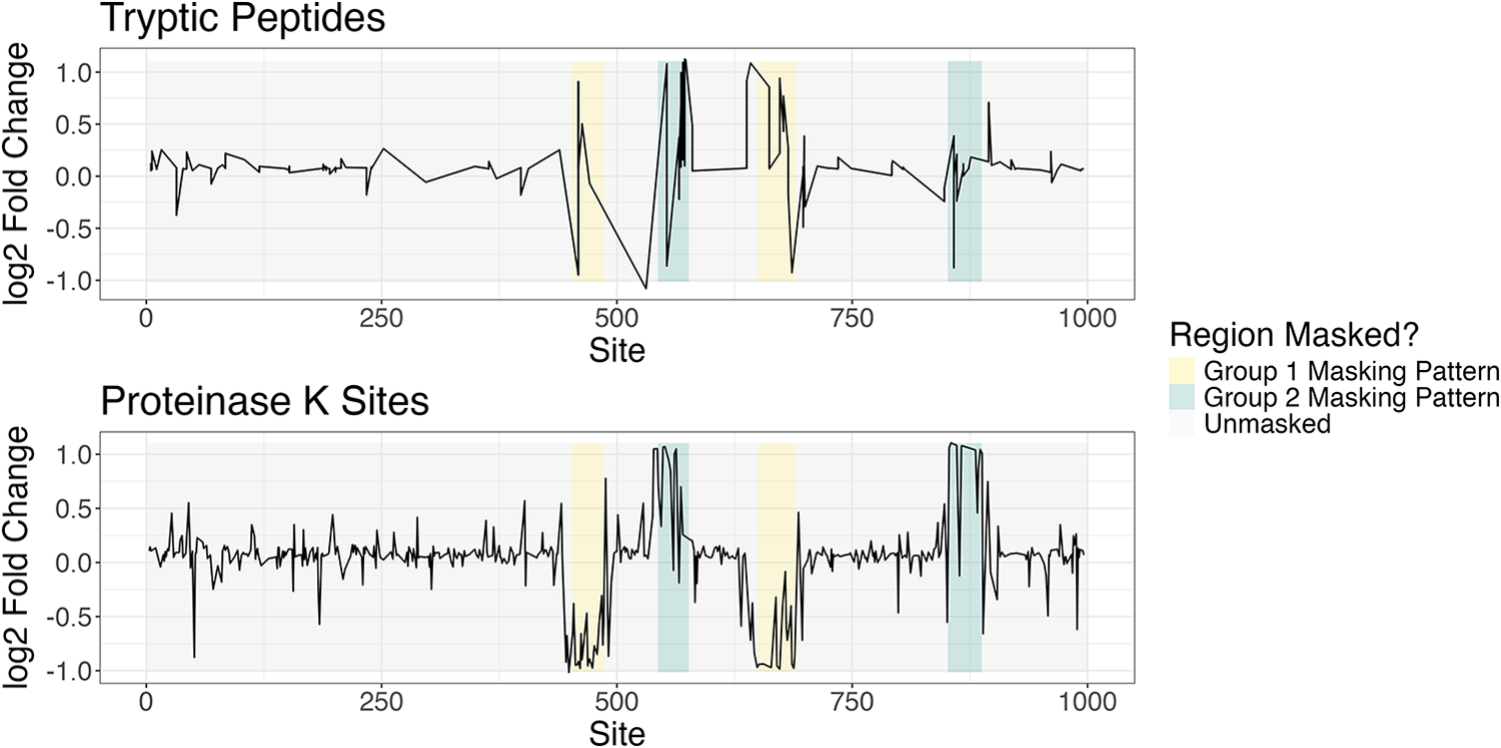
Visualization of site-level log_2_ FC with aggregated quantifications. The yellow region shows the group 1 masking pattern, the green region shows the group 2 masking pattern. The black lines are showing connected log_2_ FC calculated from digested peptides. In the upper panel is showing the log_2_ FC of tryptic sites shared by tryptic peptides. The lower panel is showing the log_2_ FC of proteinase K sites aggregated from peptides sharing the same sites.

**Figure 4:**
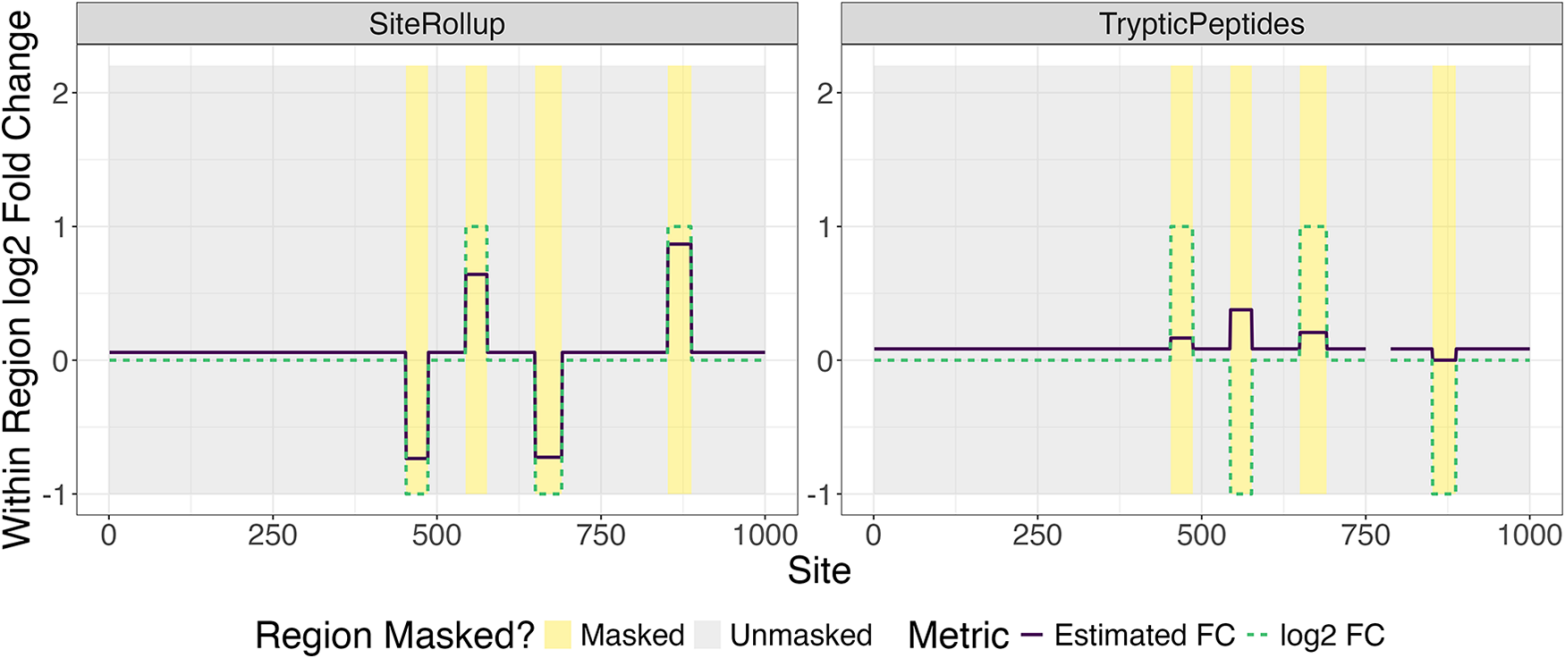
Visualization of LiP simulation evaluation. The yellow regions indicate those that were masked in one group, and not the other. The gray regions indicate regions that were not differentially masked across groups. Dotted green lines are the expected log_2_ FC for each method (ground truth), while the purple lines are the observed log_2_ FC with aggregated quantification. The left panel shows the expected log_2_ FC across the protein sequence (dotted line) versus the estimated fold change (solid green line) from the site-wise method; the right panel displays the analogous comparison for the tryptic-peptide method. Note that the true fold-change direction is opposite between the two approaches, as anticipated.

#### Site-Level Roll-Up Improves Structural Change Detection

In all LiP-MS scenarios, aggregating intensities at ProK sites outperforms the traditional tryptic-peptide approach for detecting differentially masked regions. Figure 5 summarizes these results: for regions with injected masking ratios of 2:1 or 1:2 between groups, the site-level method achieves a median RMSE of ∼0.55, compared to ∼1.0 units for tryptic-peptide aggregation – a 50% reduction in error. For nondifferential (1:1 masking) regions, tryptic aggregation shows slightly lower errors (∼0.25 vs 0.28), suggesting a modest bias in the site-level method when no true change exists. This is likely due to the higher digestion noise in ProK sites than the tryptic peptides.

**Figure 5:**
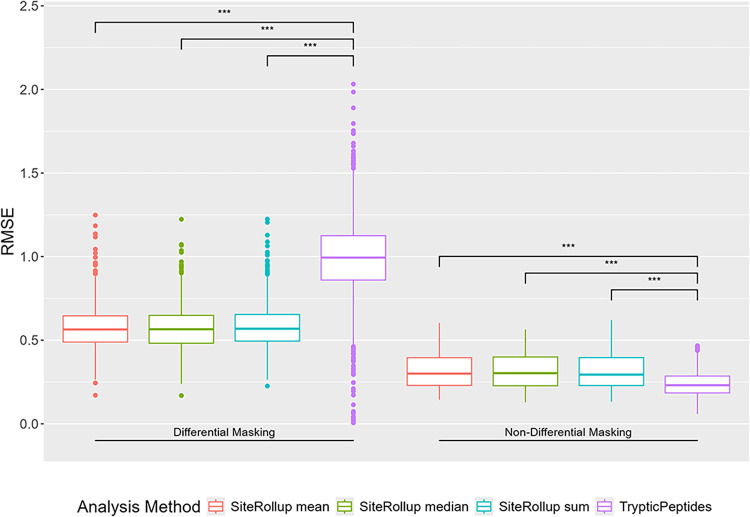
Aggregated LiP simulation results across all scenarios. Boxes with different colors show the level of errors in quantifying the log_2_ FC with different roll-up methods. Due to the high number of simulation runs, the differences between site-level and peptide-level quantification errors are significant at the 0.001 level (***) by Student’s *t-*test.

#### Consistency Across Sample Sizes and Cleavage Efficiencies

Whether simulating small cohorts (*n* = 5 per group) or larger studies (*n* = 50), site-level roll-up consistently yields lower RMSE for differential masking, with performance gains of 0.15–0.30 over tryptic peptides. Varying missed-cleavage rates for proteinase K and trypsin (0–50%) has minimal effect on the superiority of site-level aggregation, underscoring its robustness to digestion variability. More expanded comparisons are in Figures S4, S5, S6, and S7.

#### Impact of Mask and Gap Lengths

Our supplementary analysis (Figure 6) varying *λ_G_* (intermask gap) and *λ_M_* (mask size) shows that neither the distance between masked regions nor their lengths materially alters the relative performance: site-level roll-up maintains lower RMSE for true structural changes across all tested *λ_G_*/*λ_M_* combinations, confirming the generalizability of this approach. These simulations confirm that neither increasing the distance between masked regions nor enlarging mask sizes alters the overarching trend.

**Figure 6:**
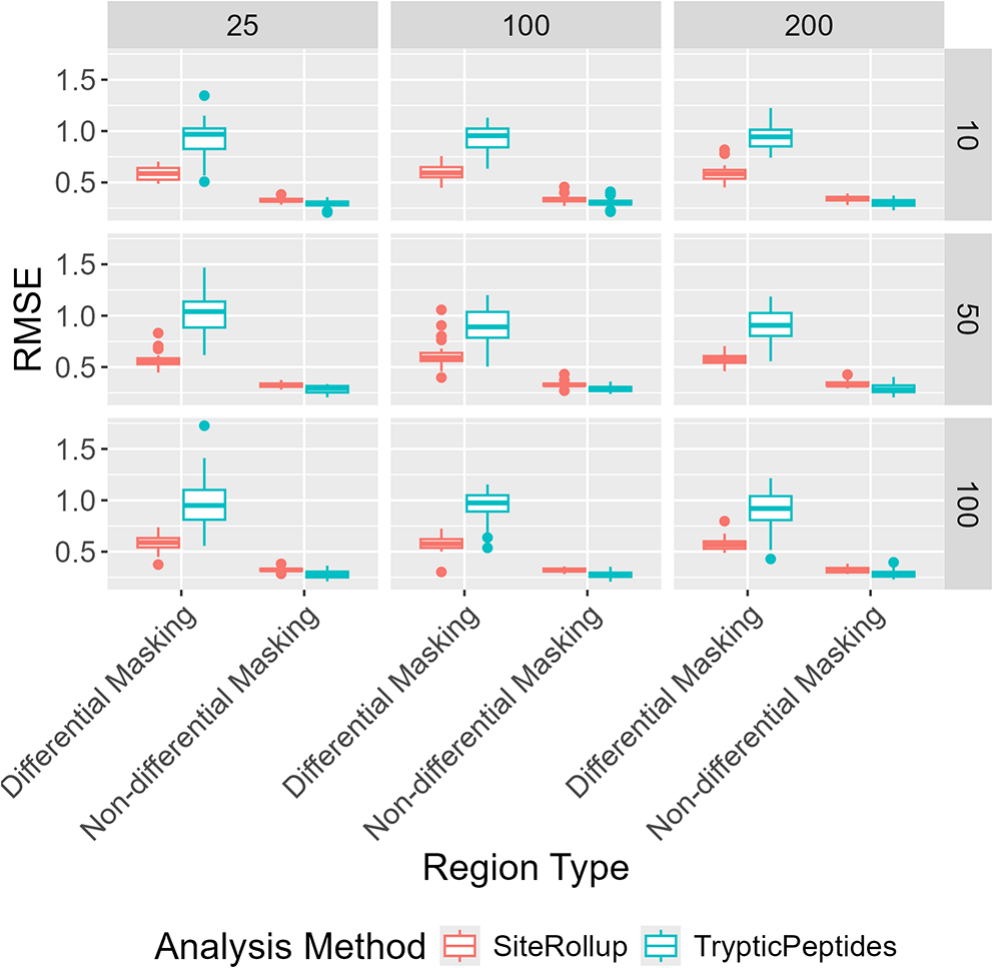
LiP simulation results when varying λ_*G*_ and λ_*M*_. The rows are showing roll-up quantification errors with different gap region lengths λ_*G*_ and the columns are showing errors with different masked region lengths λ_*M*_. These different gap and masking patterns created nine combined scenarios. In each panel, purple box is showing the ProK site roll-up while the yellow box is showing the tryptic site roll-up methods in both differential masking regions as well as nondifferential masking regions.

## Discussion

Nearly all cellular processes are regulated through structural changes, complex assembly, and dynamic modifications such as phosphorylation, acetylation, and redox modification.^21^ Dysregulated PTM and structural patterns are implicated in cancer, neurodegeneration, and metabolic disorders. Quantifying these changes in bottom-up proteomics is challenging due to the quantification at the peptide level rather than the PTM site level or structural change site level. In this study, we evaluate various roll-up strategies in two under-explored contexts: post-translational modifications (PTMs) and limited proteolysis (LiP). For PTMs, we adapt common bottom-up roll-up methods – normally applied at the protein level – to operate at the site level instead. For LiP data, we propose aggregating peptide intensities at the ProK sites and compare this site-level strategy with the conventional tryptic-peptide approach.

In principle, site-level aggregation can offer greater subprotein resolution and greater statistical robustness via the aggregation of many (noisy) peptide-level measurements. In the case of PTMs, this comes with the trade-off that one can no longer distinguish whether PTMs at sites with similar sequence positions are correlated within individual samples. Similar limitations are already present at the peptide-level because in-sample correlations among structurally close but sequentially distant PTMs are not captured at the peptide level either. Such in-sample correlations, however, only lead to differences in biological interpretation when there are heterogeneous subpopulations of a protein with mutually exclusive PTMs. We conjecture that such phenomena are rare in most systems, but more advanced experimental analysis is needed to fully asses this.

In the PTM simulations, we observed that scaling abundances using either *rollup* or *rrollup*, combined with aggregation by *mean* or *median*, consistently produced the lowest errors. Although *rollup* yielded slightly lower RMSE values, *rrollup* delivered more stable performance across all scenarios. Between *mean* and *median* aggregation, the mean generally had a marginal advantage in accuracy, but differences were small enough that we recommend choosing based on the specifics of each experiment. For the LiP simulations, aggregating to the stage 1 (proteinase K) digestion sites outperformed the tryptic-peptide method in every scenario, regardless of whether *mean* or *median* was used for roll-up. We originally hypothesized that masked-region size or inter-region distance might drive these results, but follow-up simulations – varying both masked-region lengths and gap sizes – confirmed that site-level roll-up remained superior.

Our expanded analyses demonstrate that PTM quantification benefits most from *mean* or *median* aggregation paired with *rollup* or *rrollup* scaling. In our numerical experiments, these approaches robustly handled missing data and digestion variability. Our simulations also suggest that LiP-MS structural detection is substantially improved by site-level aggregation, enhancing sensitivity to conformational changes and aligning with the strengths of current DIA-informed pipelines. These improvements align with and build upon recent methodological advances in mass spectrometry–based proteomics.

High-throughput omics analyses are inherently difficult because factors such as laboratory conditions and batch effects are hard to control or replicate, complicating data simulation. These variables are difficult or even impossible to completely account for, and thus obtaining a meaningful ground truth to use when assessing data pipelines is a challenge. In this study, we mitigate this by developing a data simulation strategy to approximate key biological processes and provide practical guidance for selecting roll-up methods. Simply assessing which method yields the most statistically significant peptides (or changes in peptides) is not sufficient on its own to conclude which method is superior. One must simultaneously assess the potential for false positives, which is challenging to accomplish experimentally. Future studies might address this challenge by implementing controlled dilution-series experiments in which biological samples are perturbed in two ways and then mixed in defined ratios. When these mixtures are analyzed via a bottom-up proteomics pipeline, the known dilution factors serve as a reliable ground truth without requiring elaborate laboratory simulations.

Overall, our work fills a gap in the literature on roll-up strategies for PTM and LiP data. We show that the same *rollup*/*rrollup* approaches used in standard bottom-up workflows – paired with mean or median aggregation – work well for site-level PTM quantification. Likewise, rolling up to proteinase K sites provides a robust alternative to tryptic-peptide analysis for LiP experiments. We hope these results inform practitioners about existing and alternative roll-up options and inspire more data-driven preprocessing guidelines for both PTM and LiP data sets.

## Supplementary Material



## Notes

Full PTM simulation study results without any induced missingness (Figure S1); full PTM simulation study results with 25% induced missingness (Figure S2); full PTM simulation study results with 50% induced missingness (Figure S3); full LiP simulation study results for the 5 samples per group scenario (Figure S4); full LiP simulation study results for the 10 samples per group scenario (Figure S5); full LiP simulation study results for the 25 samples per group scenario (Figure S6); and full LiP simulation study results for the 50 samples per group scenario (Figure S7) (PDF)
