# Supplementary material for: Aggregation Methods for Quantifying PTM and Structural Changes in Bottom-Up Proteomics
Source: J Proteome Res. 2026 May 15;25(6):3159–67. doi: 10.1021/acs.jproteome.5c00782 (PMC13248008; doi:10.1021/acs.jproteome.5c00782)
Supplement: Supplementary file 1 [file pr5c00782_si_001.pdf]

# Supplemental Material: Aggregation methods for quantifying PTM and structural changes in bottom-up proteomics

Erik D. VonKaenel,[*,†] Jordan C. Rozum,[†] Tong Zhang,[†] Kelly G. Stratton,[†] Lisa Bramer,[†] H. Steven Wiley,[‡] Wei-Jun Qian,[†] Amy C. Sims,[¶] John T. Melchior,[†] and Song Feng[*,†]

†*Biological Sciences Division, Pacific Northwest National Laboratory, Richland, WA, USA*

‡*Environmental Molecular Sciences Division, Pacific Northwest National Laboratory, Richland, WA, USA*

¶*Nuclear, Chemical, and Biological Technologies Division, Pacific Northwest National Laboratory, Richland, WA, USA*

E-mail: vonkaenelerik@gmail.com; song.feng@pnnl.gov

Figure S1: Full PTM simulation study results without any induced missingness

Figure S2: Full PTM simulation study results with 25% induced missingness

Figure S3: Full PTM simulation study results with 50% induced missingness

Figure S4: Full LiP simulation study results for the 5 samples per group scenario.

Figure S5: Full LiP simulation study results for the 10 samples per group scenario.

Figure S6: Full LiP simulation study results for the 25 samples per group scenario.

Figure S7: Full LiP simulation study results for the 50 samples per group scenario.

## Supporting Information Available

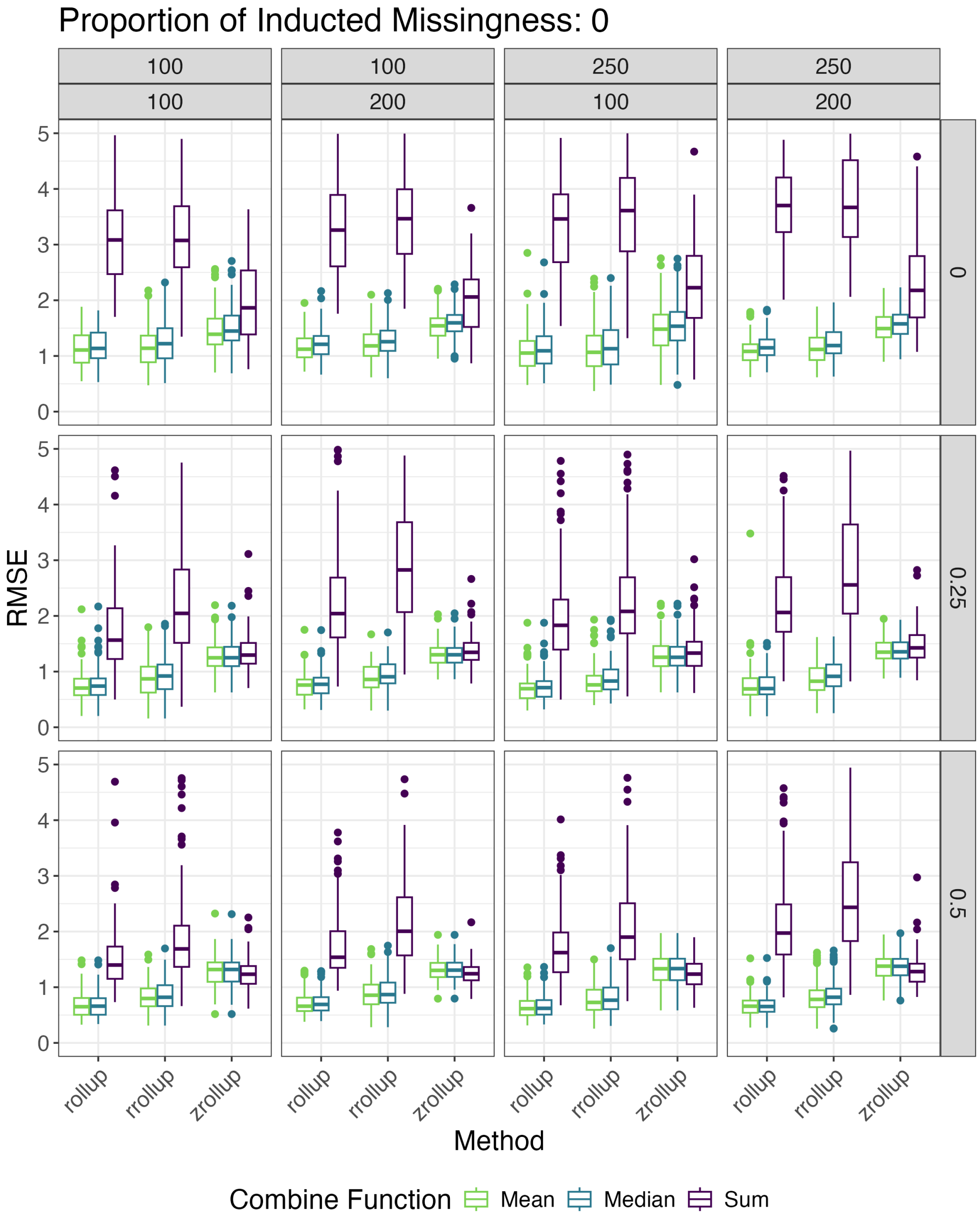


Figure S1: Full PTM simulation study results without any induced missingness. The column facets indicates the protein abundance and length, respectively, and the row facet indicates the proportion of missed tryptic sites

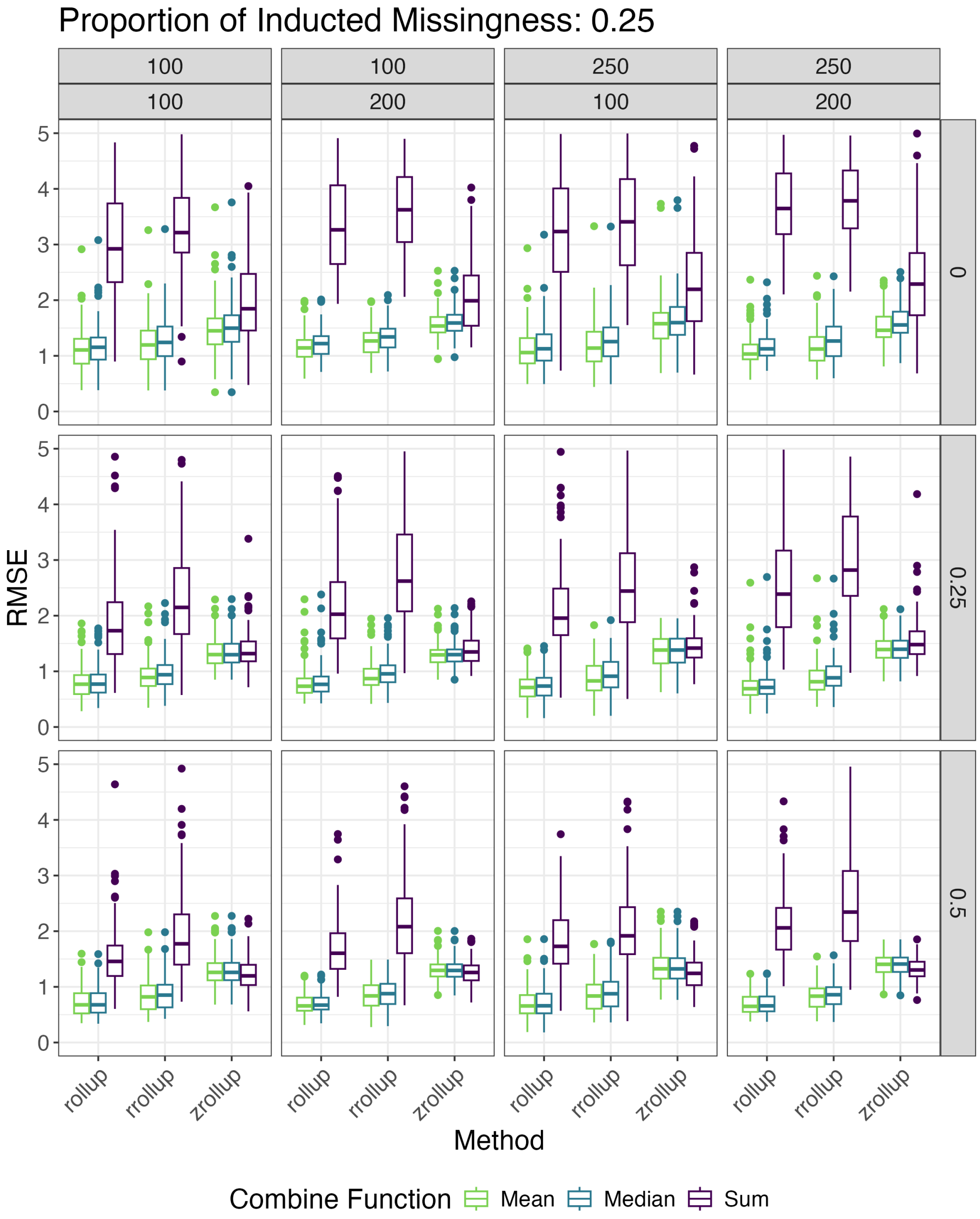


Figure S2: Full PTM simulation study results with 25% induced missingness. The column facets indicates the protein abundance and length, respectively, and the row facet indicates the proportion of missed tryptic sites

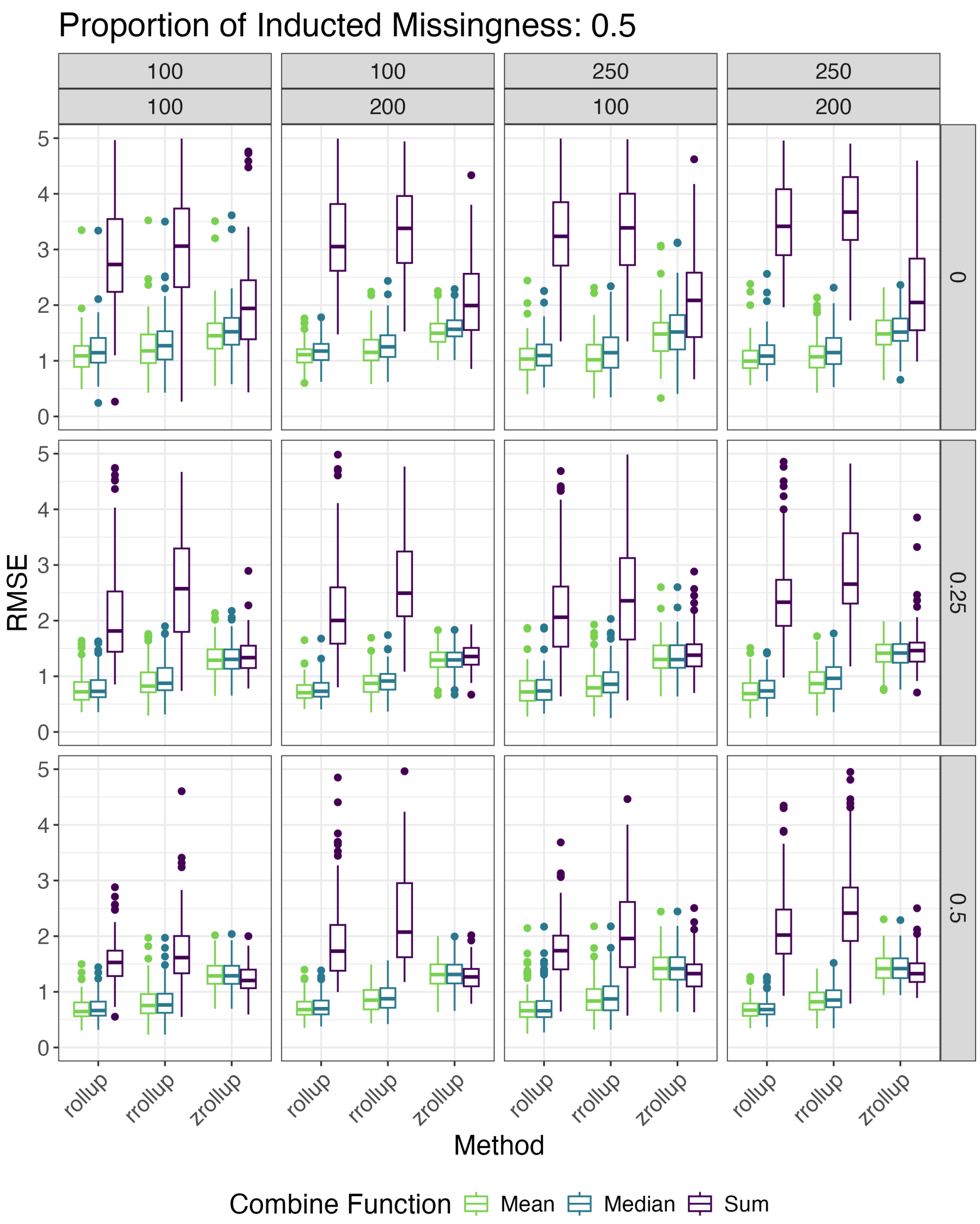


Figure S3: Full PTM simulation study results with 50% induced missingness. The column facets indicates the protein abundance and length, respectively, and the row facet indicates the proportion of missed tryptic sites

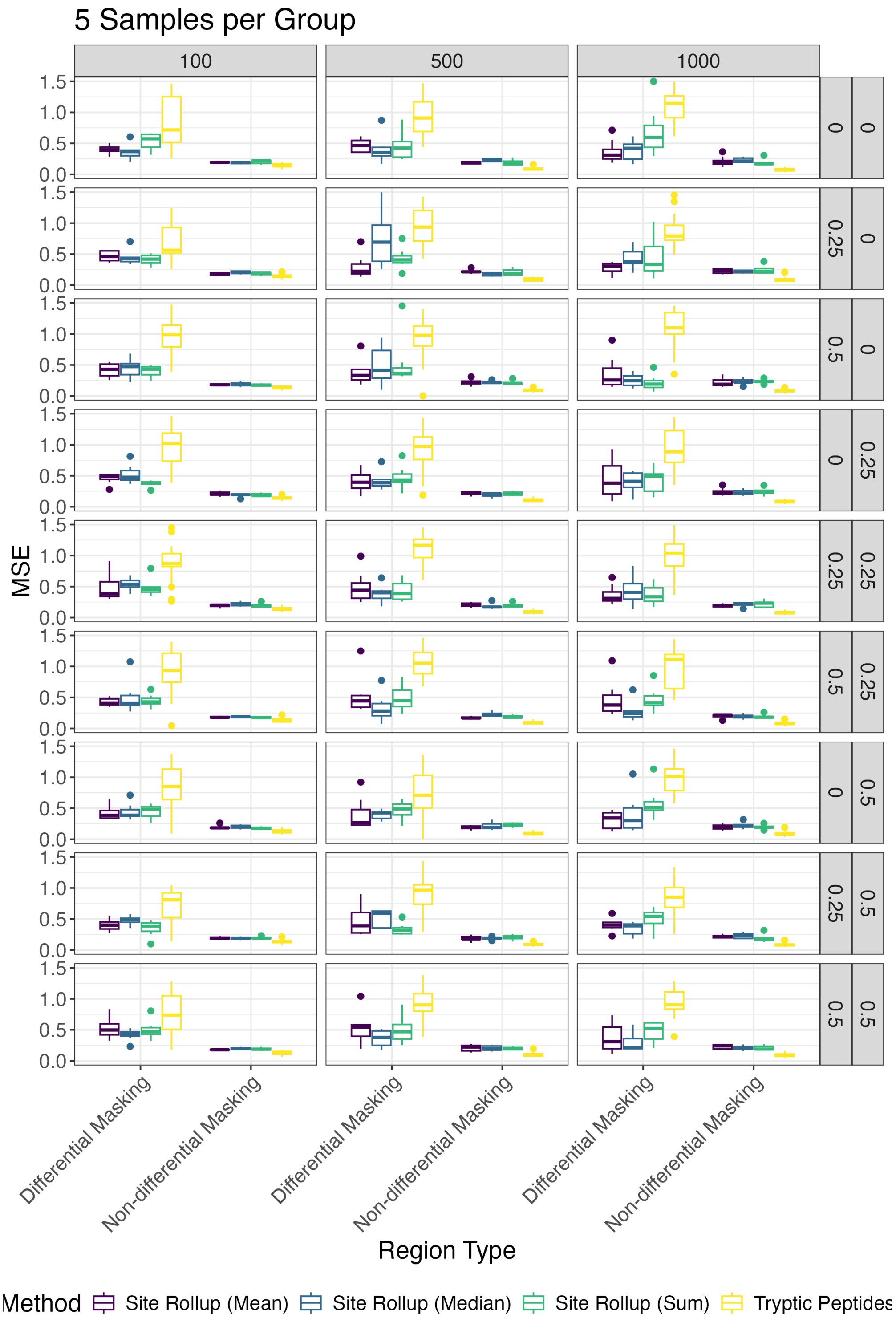


Figure S4: Full LiP simulation study results for the 5 samples per group scenario. The row facet indicates the number of protein replicates simulated, and the column facets indicate the proportion of missed tryptic and ProK sites, respectively.

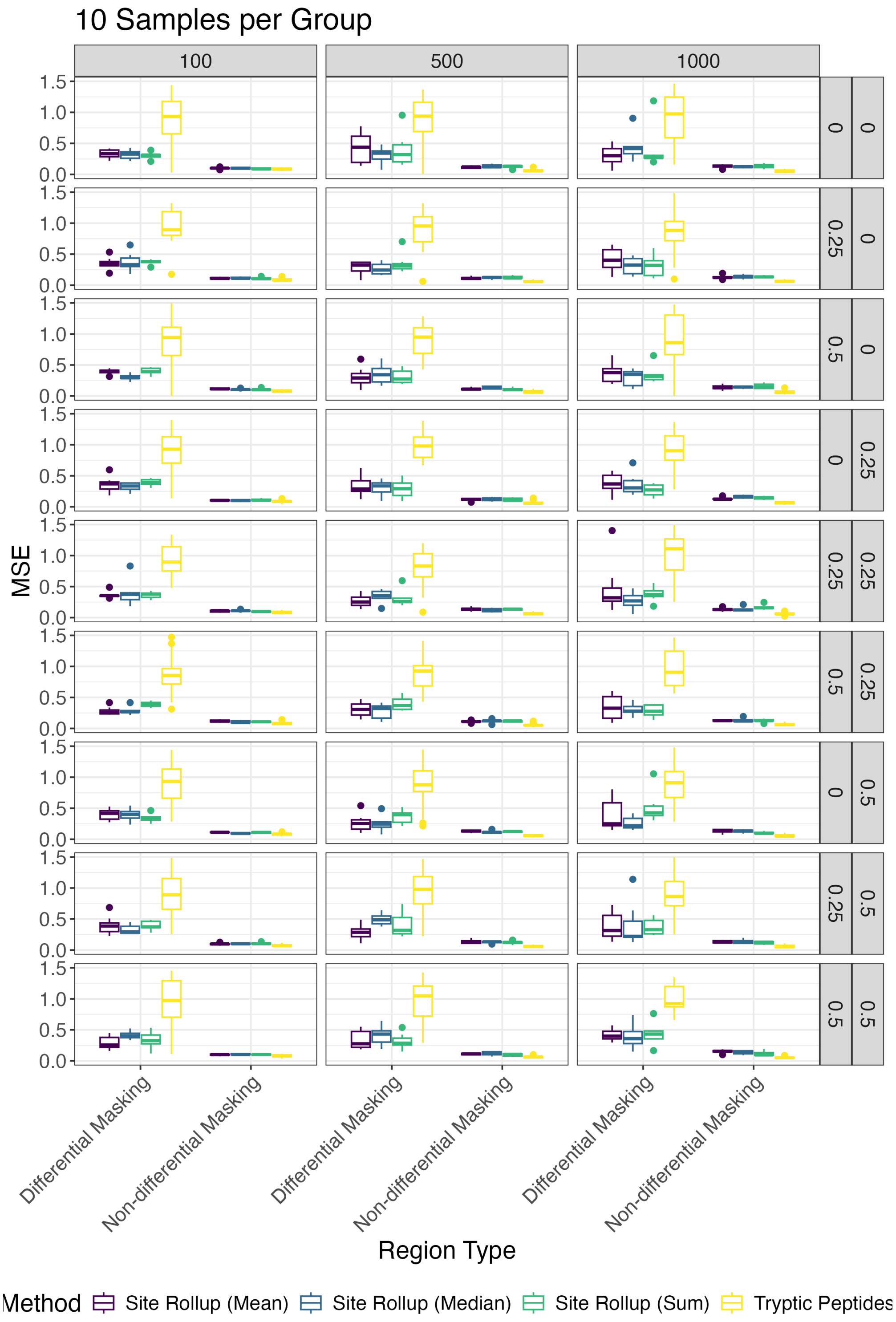


Figure S5: Full LiP simulation study results for the 10 samples per group scenario. The row facet indicates the number of protein replicates simulated, and the column facets indicate the proportion of missed tryptic and ProK sites, respectively.

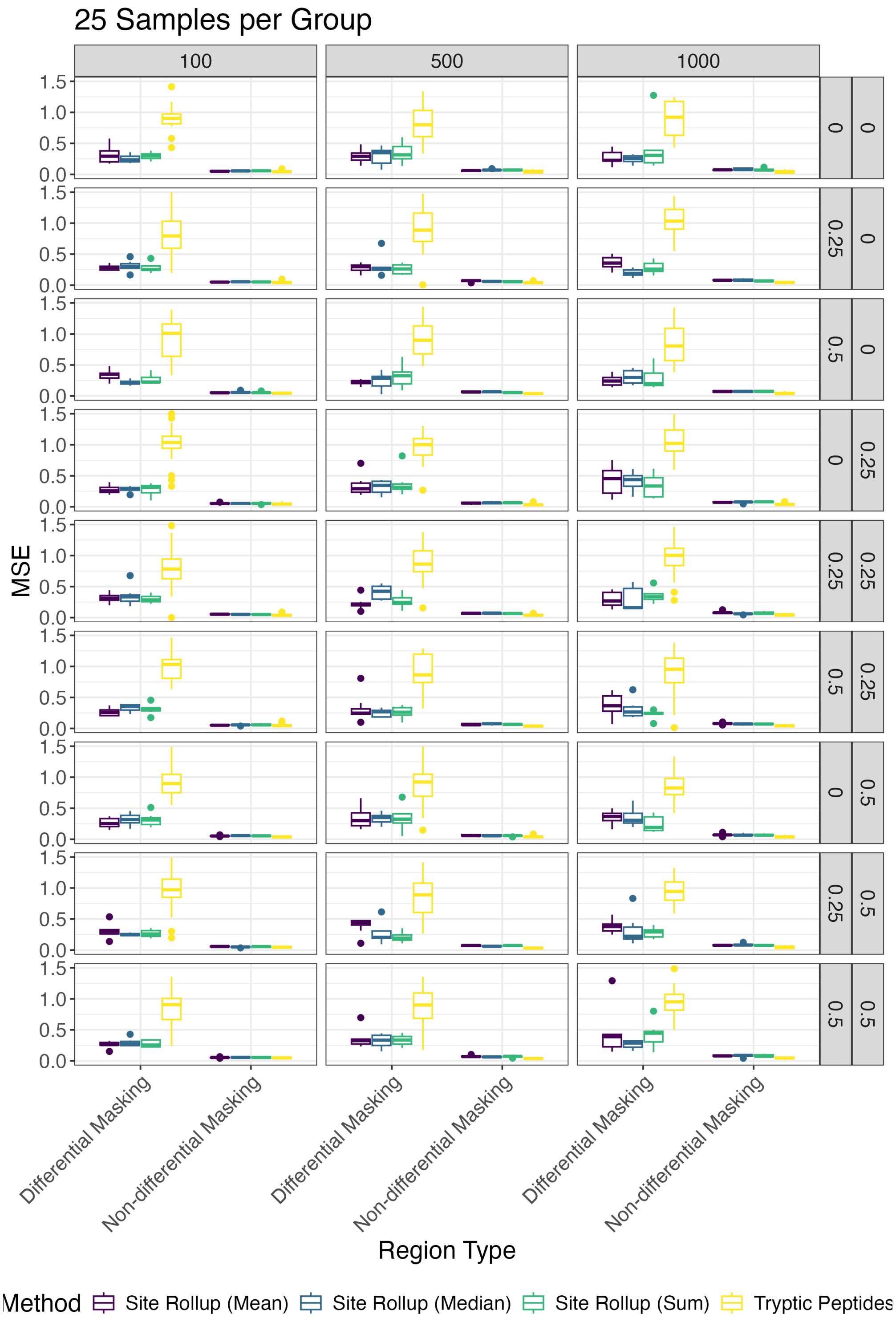


Figure S6: Full LiP simulation study results for the 25 samples per group scenario. The row facet indicates the number of protein replicates simulated, and the column facets indicate the proportion of missed tryptic and ProK sites, respectively.

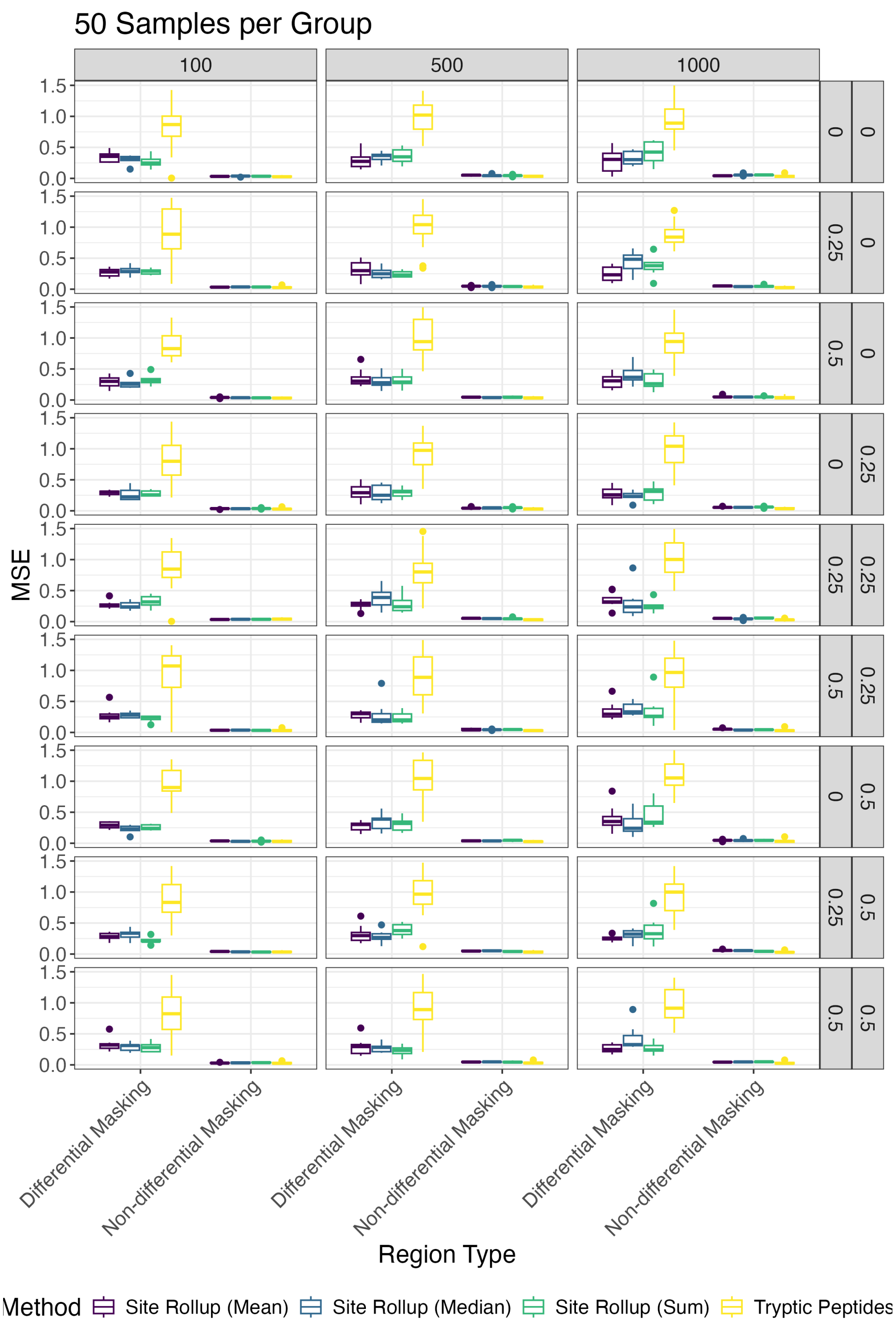


Figure S7: Full LiP simulation study results for the 50 samples per group scenario. The row facet indicates the number of protein replicates simulated, and the column facets indicate the proportion of missed tryptic and ProK sites, respectively.
